# CT-derived body composition as a predictor of supervised exercise therapy response in intermittent claudication and peripheral arterial disease: COMPOSE-PAD study protocol

**DOI:** 10.17305/bb.2026.14062

**Published:** 2026-04-15

**Authors:** Matej Pekar, Anna Pekarova, Jan Alexander Mohr, Lubomir Blaha, Marek Kantor, Barbora Ryskova, Otakar Jiravsky

**Affiliations:** 1 Complex Cardiovascular Center, Hospital AGEL Trinec-Podlesi, Trinec, Czech Republic; 2 Department of Physiology, Faculty of Medicine, Masaryk University, Brno, Czech Republic

**Keywords:** Peripheral arterial disease, intermittent claudication, supervised exercise therapy, computed tomography body composition, myosteatosis, visceral adipose tissue, sarcopenic obesity, treatment outcome prediction

## Abstract

Peripheral arterial disease (PAD) commonly presents with intermittent claudication, for which supervised exercise therapy (SET) is recommended as first-line treatment; however, real-world SET non-completion is frequent and a clinically relevant subgroup of patients fails to achieve meaningful functional improvement. No validated pre-treatment tool currently identifies these likely non-responders. This protocol describes a prospective single-centre observational cohort study designed to determine whether body composition derived opportunistically from routine computed tomography angiography (CTA) at the third lumbar vertebral level (L3), specifically skeletal muscle density and visceral adipose tissue (VAT) area, independently predicts 3-month SET outcome in patients with Fontaine IIa–IIb intermittent claudication. A total of 128 consecutive patients will be enrolled at the Complex Cardiovascular Centre, Hospital AGEL Trinec-Podlesi, Czech Republic. The primary composite outcome is treatment success at 3 months, defined as both functional improvement on standardised treadmill testing, expressed as an absolute increase of at least 50 m or a relative increase of at least 50% in maximum walking distance (MWD), and freedom from revascularisation. Treatment failure includes SET non-completion, functional non-response among programme completers, or revascularisation during follow-up irrespective of functional result. Associations between skeletal muscle density, VAT area, and treatment outcome will be examined using prespecified multivariable logistic regression adjusted for age, sex, and baseline MWD, with bootstrap internal validation. As this is a study protocol, no outcome data are yet available; enrolment is planned to begin in June 2026. This study will assess whether zero-burden metabolic phenotyping from routine CTA can help identify patients less likely to benefit from conservative management alone, but any derived model will require external multicentre validation before clinical use. ClinicalTrials.gov: NCT07433309.

## Introduction

Peripheral arterial disease (PAD) affects approximately 236 million individuals aged 25 years and older, with intermittent claudication being its most common symptomatic manifestation [1]. International clinical practice guidelines, including the 2024 American College of Cardiology/American Heart Association (ACC/AHA), 2024 European Society of Cardiology (ESC), and 2024 European Society for Vascular Surgery (ESVS) guidelines, unanimously endorse supervised exercise therapy (SET) as the first-line treatment for intermittent claudication [2–4]. Furthermore, the 2024 ESC and ESVS guidelines specify that revascularization should be deferred until conservative management has been adequately trialed [3, 4]. A Cochrane systematic review demonstrates that exercise significantly enhances maximum walking distance and functional performance compared to no exercise [5]. Additionally, a second Cochrane review confirms the superiority of SET over home-based exercise and walking advice alone [6]. A meta-analysis of 15 randomized controlled trials shows significant improvements in physical health-related quality of life and perceived walking ability with structured exercise programs, although mental health component scores were not significantly affected [7].

Despite these benefits at the population level, individual treatment responses exhibit considerable heterogeneity. In the Invasive Revascularization or Not in Intermittent Claudication (IRONIC) study, 25% of conservatively managed patients ultimately required revascularization over five years, despite achieving comparable long-term outcomes to the revascularization group. This underscores both the tolerability of conservative management and the existence of a subgroup with insufficient response [8]. Real-world SET series report non-completion rates of 40–50% [9]. Conventional clinical predictors — including ankle-brachial index (ABI), age, sex, and disease severity — do not reliably forecast the extent of functional improvement achieved with SET [10]. However, patients with lower baseline walking capacity may derive proportionally greater benefits [10]. Currently, no validated metabolic tool exists to identify, prior to therapy initiation, patients lacking the biological substrate for aerobic adaptation, making them unlikely to benefit from a sustained exercise program.

Chronic lower-limb ischemia drives progressive skeletal muscle remodeling characterized by myofiber pathology, mitochondrial dysfunction, impaired oxidative phosphorylation, signs of denervation, and intramuscular lipid infiltration, which can be detected as reduced computed tomography (CT) attenuation [11, 12]. These tissue-level alterations directly constrain the capacity for aerobic adaptation — the primary mechanism through which SET exerts its functional benefits. CT-derived skeletal muscle attenuation in Hounsfield units (HU) correlates with histologically confirmed muscle lipid content [12]. At the L3 vertebral level, single-slice CT analysis provides whole-body composition estimates that are strongly correlated with dual-energy X-ray absorptiometry (DXA; *r* ═ 0.86–0.94) [13]. Visceral adipose tissue (VAT) functions as a systemic endocrine and pro-inflammatory compartment and is independently associated with insulin resistance, atherogenic dyslipidemia, and chronic systemic inflammation, potentially impairing exercise adaptation through mechanisms beyond the ischemic limb [14]. CT angiography (CTA) is routinely performed in symptomatic PAD patients being evaluated for revascularization [2, 15], making L3 body composition extraction a low-burden, opportunistic measurement. Our group has previously demonstrated that L3 CT-derived body composition parameters independently predict outcomes in patients undergoing transcatheter aortic valve implantation (TAVI) [16], and has developed a user-friendly clinical implementation tool based on this methodology [17], providing direct biological plausibility in a comparable cardiovascular rehabilitation context.

The primary aim of this study is to determine whether L3 CT-derived skeletal muscle density and VAT area independently predict composite SET treatment success at three months in patients with Fontaine IIa–IIb intermittent claudication. Two co-primary hypotheses are tested: H1 proposes that lower skeletal muscle density per 1 standard deviation (SD) decrease will predict treatment failure; H2 posits that higher VAT area per 1 SD increase will predict treatment failure. H3, which states that the sarcopenic obesity phenotype is associated with the lowest treatment success rates across body composition phenotype groups, is designated as a secondary/descriptive hypothesis and is not included in the family-wise Bonferroni correction for the co-primary analyses. This is a prospective observational derivation cohort study, hereafter referred to as CT Body Composition as Predictor of Exercise Therapy Outcome in PAD (COMPOSE-PAD); external multicenter validation is the pre-specified and required next step before clinical translation.

## Materials and methods

### Study design and setting

This is a prospective, single-center observational cohort study (COMPOSE-PAD) conducted at the Complex Cardiovascular Centre, Hospital AGEL Trinec-Podlesi, Trinec, Czech Republic. Enrollment is anticipated to commence in June 2026 and is planned to conclude in September 2030, with primary completion expected in December 2030 (three months after the anticipated last enrollment) and study completion expected in January 2031, with long-term follow-up ending in March 2032 (18 months after the last enrollment). Long-term outcomes, including major adverse limb events (MALE), major adverse cardiovascular events (MACE), and all-cause mortality, will be assessed retrospectively after the study’s completion in January 2031. This analysis will utilize national registry data and electronic health records. The study is reported in accordance with the Standard Protocol Items: Recommendations for Interventional Trials (SPIRIT) guideline [18]. The SPIRIT checklist is provided as a supplementary file. COMPOSE-PAD is prospectively registered as an observational cohort study at ClinicalTrials.gov (NCT07433309; study type: observational; time perspective: prospective). There is no randomization, no experimental treatment allocation, and no interventional comparator.

**Table 1:** Eligibility criteria for study enrollment

**Inclusion criteria**	**Exclusion criteria**
Age ≥18 years Confirmed PAD defined by at least one of: resting ABI ≤0.90; duplex ultrasonography demonstrating ≥50% stenosis in a relevant lower limb vessel; or CTA demonstrating hemodynamically significant stenosis (≥50% diameter reduction) in a named below-aortic vessel associated with the presenting claudication territory. MRA may serve as additional confirmatory diagnostic evidence for PAD diagnosis but does not substitute for CTA eligibility; patients with MRA-only imaging lacking an eligible diagnostic CTA are excluded. “Flow-limiting” disease is defined as haemodynamically significant stenosis (≥50% diameter reduction) in a named below-aortic vessel on CTA or duplex ultrasonography, associated with the presenting claudication territory. TASC II anatomical severity classification is recorded at baseline. Fontaine stage IIa or IIb intermittent claudication Scheduled for institutional SET program Existing CTA with an L3 axial slice suitable for body composition analysis, acquired within 12 months prior to enrollment. Where multiple eligible CTAs exist within the 12-month window, the most recent scan is used. Patients without an eligible CTA within this window are excluded. Capacity to provide written informed consent	Fontaine stage III or IV PAD (critical limb-threatening ischemia) Ipsilateral lower extremity revascularization within 6 months prior to enrolment Absolute contraindication to structured exercise (e.g., unstable cardiac disease, severe pulmonary hypertension, uncontrolled arrhythmia) Cognitive impairment (MMSE < 24) Active malignancy or systemic condition expected to substantially alter body composition independently of the intervention Estimated life expectancy <12 months, assessed by the enrolling physician based on terminal malignancy, end-stage organ failure, or documented palliative care status at screening

Abbreviations: ABI, ankle–brachial index; CTA, computed tomography angiography; L3, third lumbar vertebral level; MMSE, Mini-Mental State Examination; MRA, magnetic resonance angiography; PAD, peripheral arterial disease; SET, supervised exercise therapy; TASC II, TransAtlantic Inter-Society Consensus II.

### Ethics and registration

The study protocol was approved by the ethics committee of the Hospital AGEL Trinec-Podlesi in 2022 (reference: EK 314/22) and is conducted in accordance with the Declaration of Helsinki and applicable national regulations. Written informed consent is obtained from all participants prior to any study-specific procedures. The protocol is prospectively registered at ClinicalTrials.gov [NCT07433309]. Any amendments to the protocol are reported to the ethics committee and updated in the registry before implementation.

### Eligibility criteria

Eligibility criteria are outlined in Table 1. Briefly, inclusion requires participants to be aged ≥18 years, with confirmed PAD defined by at least one of the following: resting ABI ≤0.90, duplex ultrasonography demonstrating ≥50% stenosis in a relevant lower limb vessel, or CTA demonstrating hemodynamically significant stenosis (≥50% diameter reduction) in a named below-aortic vessel associated with the presenting claudication territory, classified according to the Trans-Atlantic Inter-Society Consensus II (TASC II) anatomical severity framework and recorded at baseline. Magnetic resonance angiography (MRA) may serve as additional confirmatory diagnostic evidence for PAD diagnosis but does not substitute for CTA eligibility; patients with MRA-only imaging lacking an eligible diagnostic CTA are excluded. Participants must have Fontaine stage IIa or IIb intermittent claudication, be scheduled to participate in the institutional SET program, and have a CTA with an axial slice at the L3 vertebral level suitable for body composition analysis, acquired within 12 months prior to enrollment (the most recent eligible scan is used when multiple CTAs are available). Capacity to provide written informed consent is required. Requiring an existing diagnostic CTA selects patients who have undergone revascularization workup; the implications for external validity of this referral selection are discussed in the Limitations section. Exclusion criteria include Fontaine stage III or IV PAD; ipsilateral revascularization within six months preceding enrollment; absolute contraindication to structured exercise; cognitive impairment (Mini-Mental State Examination score <24); active malignancy expected to alter body composition independently of the exercise intervention; and estimated life expectancy <12 months, defined as the enrolling physician’s clinical judgment regarding survival likelihood based on terminal malignancy, end-stage organ failure, or documented palliative care status at screening.

### SET

The SET program follows the 2019 American Heart Association (AHA) scientific statement on optimal exercise programs for patients with PAD [19]. Each participant completes 36 supervised sessions delivered three times per week over 12 weeks, with each session lasting 60 minutes (45 minutes of structured exercise plus 15 minutes of structured patient education on vascular risk factor management and walking behavior). Treadmill exercise is initiated at 3.2 km/h on a flat gradient; participants walk until claudication pain reaches severity level 3 on a standardized four-point scale, then rest until pain subsides to level 1, repeating for five to six cycles per session.

Structured progression applies as follows: during weeks 1–4, treadmill speed increases by 0.3 km/h every two weeks to a maximum of 4.0 km/h; during weeks 5–8, treadmill grade increases by 1% every two weeks to a maximum of 3%. From week 5, resistance exercises targeting the major lower extremity muscle groups are added, beginning at two sets of 10 repetitions and progressing toward five sets at approximately 70% of one-repetition maximum. Session attendance is documented individually for each participant. Adequate adherence is defined as completion of ≥30 of 36 sessions (≥83%); non-adhering participants are retained in the primary analysis under intention-to-treat principles and analyzed separately in a pre-specified sensitivity analysis. Weekly telephone contact is maintained by a trained exercise physiologist to monitor symptoms and reinforce adherence. Participation in any other formal structured exercise program or rehabilitation intervention during the 12-week SET period is not permitted; habitual daily physical activity and unsupervised walking are allowed.

### CT body composition analysis

Body composition analysis is derived from a single axial CT slice at the L3 vertebral level. The L3 landmark is defined as the mid-vertebral body of L3, as identified on the scout image, in accordance with the methodology established by Paris et al. (2020) [20]. The L3 landmark serves as a validated systemic whole-body phenotyping tool, though it does not directly measure ischaemia-specific lower limb myopathology. This landmark demonstrates strong correlations with DXA-derived lean and fat mass (r = 0.86–0.94) [13]. Segmentation is conducted using AutoMATiCA [20], a U-Net convolutional neural network validated in multi-morbid clinical cohorts, including critically ill patients, cancer patients, those with liver cirrhosis, and organ donors. However, its performance in PAD-specific imaging, particularly in cases with prevalent vascular calcification, has not been independently verified, a limitation that will be addressed. AutoMATiCA achieves Dice similarity coefficients exceeding 0.97 for skeletal muscle, VAT, and subcutaneous adipose tissue (SAT), while achieving 0.90 for intramuscular adipose tissue (IMAT). The lower accuracy of IMAT segmentation is acknowledged, thus designating it as an exploratory rather than a primary variable in this protocol. The processing time for segmentation is approximately 350 milliseconds per scan.

Routine CTA at our center employs helical (spiral) acquisition. The L3 body composition slice is a reformatted axial image derived from the existing helical dataset, thereby incurring no additional radiation exposure.

Compartment boundaries are determined using a combination of HU thresholds and spatial-mask criteria. Skeletal muscle area is segmented within the muscle fascia boundary using an HU threshold of --29 to +150 HU; structures exceeding 150 HU, such as calcified plaque, are excluded. VAT area is segmented within the peritoneal cavity using an HU range of --150 to --50 HU. SAT area is delineated exterior to the body wall with an HU range of --190 to --30 HU. IMAT area is segmented within the muscle fascia mask, external to individual muscle boundaries, using the same HU range of --190 to --30 HU; SAT and IMAT are differentiated solely by their respective spatial masks. Skeletal muscle density is recorded as the mean attenuation value in HU across the segmented muscle compartment. Detailed parameters, thresholds, and analytical roles are presented in Table 2.

**Table 2:** Definitions, thresholds, and analytical roles of CT body composition parameters

**Parameter**	**HU range**	**Spatial mask**	**Units**	**Threshold**	**Analytical role**
Skeletal muscle density	--29 to +150	Within muscle fascia	HU (mean)	Cohort-derived; exploratory only (Youden-index derivation; see Section 2.6); not used in primary model	Primary predictor (H1; continuous z-score in primary model); predictor for secondary sarcopenic obesity classification (H3; secondary/descriptive hypothesis)
VAT area	--150 to --50	Within peritoneal cavity	cm^2^	Cohort-derived, sex-specific; exploratory only (see Section 2.6); not used in primary model	Primary predictor (H2; continuous z-score in primary model); predictor for secondary sarcopenic obesity classification (H3; secondary/descriptive hypothesis)
SMI	--29 to +150	Within muscle fascia	cm^2^/m^2^		Covariate in pre-specified secondary expanded model only (not included in primary model); exploratory predictor of sarcopenia by mass
SAT area	--190 to --30	Exterior to body wall	cm^2^	—	Covariate in pre-specified secondary expanded model only (not included in primary model)
IMAT area	--190 to --30	Within fascia, exterior to individual muscle boundaries	cm^2^	—	Exploratory variable

Abbreviations: CT, computed tomography; HU, Hounsfield units; IMAT, intramuscular adipose tissue; SAT, subcutaneous adipose tissue; SMI, skeletal muscle index; VAT, visceral adipose tissue.

Quality control procedures include verification that calcified plaque is excluded from the muscle compartment, flagging scans with an inter-compartment boundary coefficient of variation exceeding 35% for consensus adjudication by two independent analysts, and benchmarking inter-analyst agreement on the first 20 scans of the series prior to full enrollment. This is assessed using Bland-Altman analysis and the intraclass correlation coefficient (ICC). A pre-specified ICC threshold of ≥0.90 is required for both skeletal muscle density and VAT area before proceeding to full enrollment. If the ICC falls below 0.90 on the 20-scan benchmark, the segmentation protocol will be reviewed, and analysts retrained; an additional benchmark of 10 scans will be conducted before full enrollment begins. Baseline body composition (T0) is derived from existing diagnostic CTA scans, with the most recent scan selected when multiple eligible CTAs are available within a 12-month window. A standardization and inter-scanner variability policy will be documented prior to the first enrollment and applied throughout the multi-year enrollment period; any scanner upgrades or protocol changes will also be documented and assessed for their potential impact on HU consistency.

#### CT acquisition protocol

CTA at our center is performed on a Siemens SOMATOM Force (Siemens Healthineers, Erlangen, Germany) utilizing helical acquisition with standard parameters: tube voltage of 100–120 kVp; tube current of 200–350 mAs (with automated exposure control); and slice thickness of ≤3 mm (reconstruction interval of 1.0–1.5 mm). Contrast-enhanced studies utilize iodinated low-osmolality contrast media (iohexol or equivalent), with a weight-based dose calculation of 1.5 mL/kg, capped at 150 mL. Bolus tracking is employed for timing the arterial phase (trigger threshold of 100 HU in the descending aorta, with a scan delay of 6–8 seconds post-trigger). Body composition HU measurements are conducted on post-contrast arterial-phase images, consistent with methods used in prior validation studies for this cohort [16]. Artificial intelligence (AI)-based noise reduction is not currently implemented in the clinical reconstruction pipeline. Images are reconstructed using a soft-tissue kernel (Siemens Br40 or equivalent), a parameter that remains constant across all study acquisitions and directly influences absolute HU values in body composition analysis. Iterative reconstruction techniques, specifically Sinogram Affirmed Iterative Reconstruction (SAFIRE) at strength 3, are employed, or an equivalent method is utilized based on the institutional protocols at the time of image acquisition. The imaging matrix is configured to 512 × 512, with a field of view ranging from 350 to 450 mm, tailored to the patient’s habitus, yielding a pixel size of approximately 0.68 to 0.88 mm. Both parameters are documented in the study log, and any deviations from standard settings are recorded and assessed for their impact on HU calibration. Post-acquisition processing for body composition analysis employs the AutoMATiCA neural network [20] applied to the single reformatted axial slice at the L3 mid-vertebral body level. No additional post-processing beyond segmentation and HU measurement is performed. All scans are acquired and reconstructed using a single standardized institutional protocol; any deviations are documented in the study log and evaluated for their potential impact on HU calibration. Inter-scan variability arising from the multi-year enrollment period will be monitored by including 10 phantom or repeated-scan quality controls annually, with HU drift quantified and reported.

### Body composition parameters and sarcopenic obesity classification

Body composition parameters, HU thresholds, spatial masks, derivation, units, and analytical roles are specified in Table 2. In the primary model (H1 and H2), skeletal muscle density and VAT area are entered as continuous standardized z-scores (mean 0, SD 1), facilitating direct comparison of effect estimates on a common scale. Nonlinearity of each continuous predictor is assessed using restricted cubic splines (three to five knots, selected based on the Akaike Information Criterion). The primary predictor for H1 is skeletal muscle density (HU). The exploratory classification threshold for low skeletal muscle density (myosteatosis) will be derived from this cohort by maximizing the Youden index (sensitivity + specificity -- 1) against the binary primary composite outcome, implemented using the cutpointr R package with 1000-sample bootstrap confidence intervals (CIs). This threshold derivation is explicitly designated as exploratory and hypothesis-generating; it is not included in the primary predictive analysis, which utilizes the continuous z-score. Derived thresholds are subject to substantial optimism in a derivation cohort and require external validation before clinical application. This approach is preferred over adopting thresholds from the oncological literature—specifically, the body mass index (BMI)-stratified values reported by Martin et al. [21] (41 HU for BMI < 25 kg/m^2^; 33 HU for BMI ≥ 25 kg/m^2^) or the Health, Aging, and Body Composition (ABC) Study mid-thigh attenuation reference [22]—as neither has been validated in a PAD population, and the biological relationship between muscle lipid infiltration and aerobic exercise adaptation in chronic limb ischaemia may differ significantly from oncological or elderly community contexts. Sensitivity analyses applying the thresholds from Martin et al. (33 HU and 41 HU; see Table 3, sensitivity analyses #8 and #9) are pre-specified to facilitate direct comparison with the cohort-derived threshold.

**Table 3:** Pre-specified sensitivity and secondary analyses

**#**	**Analysis**	**Rationale**
1	Alternative composite threshold: ≥40% relative MWD gain	Tests sensitivity of the primary outcome to a more conservative functional threshold
2	Alternative composite threshold: ≥60% relative MWD gain	Tests sensitivity to a more stringent functional threshold
3	Alternative composite threshold: ≥100 m absolute MWD gain	Tests sensitivity to an absolute rather than relative distance criterion
4	Functional improvement component only (no revascularization criterion)	Isolates the predictive relationship with functional adaptation. Mandatory pre-specified secondary analysis; reported in all primary publications regardless of composite outcome result.
5	Revascularisation-free survival component only	Isolates the predictive relationship with intervention requirement. Mandatory pre-specified secondary analysis; reported in all primary publications regardless of composite outcome result.
6	Restriction to guideline-concordant revascularisation events only	Removes patient-preference-driven events to reduce confounding; specifically examines discordant cases (functional improvement achieved but revascularisation occurred)
7a	Four-predictor model: muscle density, VAT, age, sex (excluding baseline MWD)	Robustness check with an EPV approximately 10; tests stability of primary model estimates when baseline MWD is excluded
7b	Three-predictor model (muscle density, VAT, age; excluding sex)	Robustness check with an EPV approximately 13–14
8	Alternative muscle density threshold: 33 HU	Tests lower threshold sensitivity for myosteatosis classification (Martin et al. [21] value for BMI ≥ 25 kg/m^2^); see also Section 2.6
9	Alternative muscle density threshold: 41 HU [Martin et al. [21], BMI < 25 kg/m^2^ stratum]	Tests higher threshold sensitivity for myosteatosis classification; see Section 2.6
10	Alternative VAT threshold: --20 cm^2^ (110 cm^2^ M; 80 cm^2^ F)**†**	Tests sensitivity to a more conservative VAT threshold
11	Alternative VAT threshold: +20 cm^2^ (150 cm^2^ M; 120 cm^2^ F)**†**	Tests sensitivity to a more stringent VAT threshold
12	Adherent subgroup (≥30/36 sessions completed)	Per-protocol analysis to assess treatment-specific effects
13	30% treatment failure rate scenario (approximately 30 failures)	Reports model performance and power under a more optimistic clinical assumption
14	Expanded confounding model: primary five predictors plus ABI, smoking status, diabetes, Clinical Frailty Scale score, TASC II classification, SMI, SAT	Pre-specified secondary expanded model; EPV approximately 3–4 (acknowledged limitation); designated as robustness sensitivity analysis; tests whether primary predictor associations are independent of additional clinical confounders
15	Complete-case sensitivity analysis	Comparison with the primary MICE-based analysis to assess the impact of the missing-at-random assumption; all cases with complete data on the five primary predictors and primary outcome are included

† Reference VAT thresholds utilized for sensitivity offsets are 130 cm^2^ for men [23] and 100 cm^2^ for women [24]. Sensitivity bounds are tested with ±20 cm^2^ offsets around these reference values. Abbreviations: ABI, ankle–brachial index; BMI, body mass index; EPV, events per variable; F, female; HU, Hounsfield units; M, male; MICE, multiple imputation by chained equations; MWD, maximum walking distance; SAT, subcutaneous adipose tissue; SMI, skeletal muscle index; TASC II, TransAtlantic Inter-Society Consensus II; VAT, visceral adipose tissue.

The primary predictor for H2 is VAT area (cm^2^). Exploratory sex-specific classification thresholds for elevated VAT will be derived separately for men and women by maximizing the Youden index against the binary primary composite outcome, using the same cutpointr bootstrap framework. This threshold derivation is explicitly designated as exploratory and hypothesis-generating, with the continuous z-score employed in the primary model. This approach is preferred over adopting general-population thresholds (>130 cm^2^ in men and >100 cm^2^ in women) from weight-loss and metabolic studies in non-PAD populations [23, 24], as the VAT level at which pro-inflammatory signaling meaningfully constrains exercise adaptation in claudicants has not been established. Sensitivity analyses applying the literature-derived VAT thresholds [23, 24] are pre-specified in Table 3 (sensitivity analyses #10 and #11). The biological rationale for VAT as a cardiometabolic risk marker is discussed in the Introduction [14]. The skeletal muscle index (SMI; cm^2^/m^2^) and SAT area are designated as covariates in the pre-specified secondary expanded model only; they are excluded from the primary model (see Section 2.12). IMAT area is analyzed as an exploratory variable. H3—sarcopenic obesity—is operationally defined as a secondary/descriptive hypothesis by the co-occurrence of skeletal muscle density below the cohort-derived exploratory threshold and VAT area above the cohort-derived sex-specific exploratory threshold, resulting in a four-group phenotype classification: normal (reference group), isolated low muscle density, isolated elevated VAT, and sarcopenic obesity. H3 is not included in the family-wise Bonferroni correction.

### Primary outcome

The primary outcome is a composite endpoint of treatment success at three months, defined by the fulfillment of both criteria: (a) functional improvement, characterized by an absolute increase in maximum walking distance (MWD) of ≥50 meters or a relative increase of ≥50% from baseline, as measured during standardized treadmill testing at a fixed speed of 3.2 km/h on a flat (0% grade) surface. This protocol is independent of the progressive speed and grade increments applied during SET training sessions. The treadmill test concludes when the participant reaches claudication pain level 3 on a standardized four-point scale or a maximum duration of 20 minutes, whichever occurs first. Pain-free walking distance (PFWD) is defined as the distance at which the participant first reports the onset of claudication pain (level 1). A standardized familiarization trial is conducted during the baseline assessment visit prior to recording the baseline MWD; participants who have undergone standardized treadmill testing within the previous 12 months may have the familiarization trial waived at the discretion of the principal investigator, with documentation of the rationale. The 50-meter absolute threshold aligns with improvement benchmarks established in the 2019 AHA scientific statement on optimal exercise programs for PAD [19]. However, no formally validated minimal clinically important difference for MWD has been established specifically for intermittent claudication. For comparison, the 6-minute walk test (6MWT) has an established minimal clinically important difference of approximately 50 meters in PAD [25]. Treadmill testing is preferred in this protocol as it directly measures claudication-limited MWD and PFWD under standardized conditions, and the progressive-challenge treadmill protocol aligns with SET training conditions. The 2-minute walk test is included as a secondary outcome to enhance cross-study comparability with research utilizing time-limited walking assessments.

The composite endpoint comprises four pre-specified outcome combinations, addressed as follows: The primary analysis of treatment failure includes three specific scenarios: (i) program non-completion, defined as failure to attend the requisite number of SET sessions, regardless of functional outcome; (ii) functional non-response among program completers, characterized by completion of the program without achieving the MWD threshold at three months (T1); and (iii) revascularization occurring within the three-month follow-up period, irrespective of functional outcome. This approach aligns with the protocol’s objective of identifying patients capable of deriving sufficient benefit from conservative management alone. The rationale for classifying revascularization as treatment failure, even when accompanied by functional improvement, is that such cases indicate the conservative management pathway was not maintained. Sensitivity analysis #6 (Table 3) examines the subset of guideline-concordant revascularization events separately.

The four pre-specified outcome combinations are: (1) functional improvement achieved AND revascularization-free = treatment success; (2) functional improvement not achieved AND revascularization occurred = treatment failure; (3) functional improvement achieved BUT revascularization occurred = classified as treatment failure in the primary analysis; this discordant case type (functional improvement with revascularization) will be specifically examined in sensitivity analysis #6 (Table 3), restricting to guideline-concordant revascularization events only, to assess whether revascularization was guideline-concordant or driven by patient preference; (4) functional improvement not achieved AND revascularization-free = treatment failure. Failure to meet either criterion (b) or the MWD threshold in (a) constitutes treatment failure.

MWD is assessed by a trained physiotherapist blinded to body composition data. Revascularization events are independently adjudicated by a vascular surgeon blinded to body composition data and predictor values, classified as: guideline-concordant (meeting ESVS 2024 criteria for intervention in claudication); patient-preference driven (not meeting objective intervention criteria); or indeterminate. For this study, guideline-concordant revascularization is defined according to ESVS 2024 criteria [4], requiring all three of the following: (a) failure to achieve meaningful functional improvement after an adequate SET trial (≥12 weeks); (b) lifestyle-limiting claudication symptoms refractory to conservative management; and (c) documented hemodynamic deterioration via ABI measurement. Events that do not meet all three criteria are classified as non-concordant or patient-preference driven. Pre-specified sensitivity analyses include composite threshold alternatives (≥40% and ≥60% relative, and ≥100 meters absolute MWD gain) and separate analyses of functional improvement and revascularization outcomes independently.

### Secondary and exploratory outcomes

Secondary outcomes, evaluated at baseline (T0) and three months (T1), include: standardized treadmill MWD and PFWD; absolute and relative change in MWD from baseline to T1 (continuous secondary outcomes); 30-second chair stand test repetitions; bilateral handgrip dynamometry (kg); 2-minute walk test distance; and Walking Impairment Questionnaire (WIQ) domain scores. Exploratory outcomes assessed at 6, 12, and 18 months—such as late revascularization, MALE, MACE, and all-cause mortality—are obtained through structured telephone follow-up, medical records review, and registry linkage. Given the anticipated low event counts during extended follow-up in this cohort, exploratory outcomes will be reported descriptively and interpreted as hypothesis-generating only.

### Assessment schedule

The schedule of enrollment, interventions, and assessments is detailed in Table 4.

**Table 4:** Schedule of enrollment, interventions, and assessments (SPIRIT SoE)

**Assessment**	**Screening (T-1)**	**Baseline (T0)**	**3 months (T1)**	**6, 12, 18 months (T2)**	**Withdrawal**
**Enrollment**	✓				
Eligibility screening	✓				
Informed consent	✓				
**Interventions**					
SET (36 sessions over 12 weeks)		→→→	→ end		
**CT body composition (L3)**					
Existing CTA analysis (T0)		✓			
Exploratory biomarker sampling — all participants (adiponectin, leptin, IL-6; samples stored at T0 and T1; interim assay after the first 40 participants complete follow-up)	✓		✓		
**Primary outcome**					
Composite SET outcome (MWD + revascularization)			✓		
**Secondary outcomes**					
Standardized treadmill MWD and PFWD		✓	✓		✓
30-s chair stand test		✓	✓		
Bilateral handgrip dynamometry		✓	✓		
2-minute walk test		✓	✓		
WIQ domain scores		✓	✓		✓
**Exploratory outcomes**					
MALE, MACE, all-cause mortality				✓	
**Clinical and metabolic data**					
Fasting metabolic panel (glucose, HbA1c, lipids, hsCRP)		✓	✓		
CFS and SPPB		✓	✓		
Adverse events monitoring			→→→	✓	

✓ ═ assessment performed at this time point; → ═ ongoing intervention across this period (arrow span indicates duration); SET delivered over 36 sessions across 12 weeks. Abbreviations: CFS, Clinical Frailty Scale; CT, computed tomography; CTA, computed tomography angiography; HbA1c, glycated haemoglobin; hsCRP, high-sensitivity C-reactive protein; IL-6, interleukin-6; L3, third lumbar vertebral level; MACE, major adverse cardiovascular events; MALE, major adverse limb events; MWD, maximum walking distance; PFWD, pain-free walking distance; SET, supervised exercise therapy; SoE, schedule of enrolment, interventions, and assessments; SPIRIT, Standard Protocol Items: Recommendations for Interventional Trials; SPPB, Short Physical Performance Battery; WIQ, Walking Impairment Questionnaire.

Withdrawal visit procedure: Participants who withdraw from the study prior to the three-month primary outcome assessment are invited to attend a dedicated withdrawal visit within two weeks of discontinuing the SET program. During this visit, standardized treadmill MWD and PFWD are recorded, and the WIQ is administered. These data will be included in the intention-to-treat analysis. Reasons for withdrawal are documented using a structured withdrawal form.

### Sample size

Sample size is determined primarily by the precision required for the primary model’s discrimination statistic [26, 27]. A minimum of 40 treatment failures is targeted for the five-predictor primary logistic regression model (muscle density, VAT area, age, sex, baseline MWD), yielding an events per variable (EPV) ratio of approximately 8. This EPV is recognized as modest, providing the primary rationale for the conservative four-predictor robustness check (Table 3, sensitivity analysis #7a). A supplementary power calculation confirms approximately 80% power to detect an odds ratio ≥2.0 per 10 HU decrease in skeletal muscle density (α ═ 0.05, two-tailed).

Assuming a treatment failure rate of 40%, reflecting the program non-completion component of the 40–50% failure rate reported in real-world European SET series [9] and a local institutional audit (approximately 38%); this 40% assumption captures both program non-completion and early revascularization combined, without separately modeling the functional non-response rate among completers. Accounting for 22% attrition from dropout and protocol non-completion, 128 patients must be enrolled to achieve 100 evaluable participants with 40 treatment failures. The IRONIC trial [8], which demonstrated that a significant proportion of claudicants managed conservatively required revascularization during follow-up, supports the clinical relevance of this failure rate, although it was not designed to quantify SET-specific non-response. A pre-specified sensitivity analysis reports model performance under a 30% failure rate scenario (approximately 30 failures, approximately 70% power). All precision-related conclusions are explicitly framed within the derivation cohort boundaries; external validation is necessary before any clinical translation.

### Blinding

This is an observational cohort study without randomization or treatment allocation. The CT analyst performing body composition segmentation is blinded to participant clinical outcomes. The vascular surgeon adjudicating revascularization events is blinded to body composition data and predictor values throughout the follow-up period. Disagreement resolution procedure: In cases where the adjudicating vascular surgeon cannot reach a clear classification (concordant versus non-concordant revascularization), a second independent vascular surgeon reviews all relevant clinical documentation while remaining blinded to body composition data. If disagreement persists after an independent second review, the case is resolved through consensus discussion between both adjudicators; the process and final classification are documented in the study log. Indeterminate cases after consensus are classified as non-concordant for the primary analysis and concordant in a pre-specified sensitivity analysis. Given the observational, non-interventional nature of this study and the absence of experimental treatment allocation, no independent data monitoring committee is established; no interim analyses or stopping rules are pre-specified.

### Statistical analysis

**Analytical hierarchy:** In all primary analyses (H1 and H2), skeletal muscle density and VAT area are treated as continuous standardized z-scores (mean = 0, SD = 1). This approach serves as the foundation for all inferential conclusions. All classification thresholds—whether derived from cohorts via Youden index maximization (cutpointr) or from literature pertaining to non-PAD populations (Martin et al. [21]; general population VAT reference values [23, 24])—are established post-hoc and are explicitly designated as exploratory and hypothesis-generating. These thresholds do not influence the primary predictive model and necessitate external validation in an independent PAD cohort prior to any clinical application. This distinction is consistently maintained throughout the statistical analysis section.

All analyses are conducted using International Business Machines (IBM) Statistical Package for the Social Sciences (SPSS) Statistics, version 30 (IBM Corp., Armonk, NY) and R version 4.3 (R Foundation for Statistical Computing, Vienna, Austria; packages: rms, cmprsk, mice, boot, cutpointr, survminer). The complete statistical analysis plan is pre-registered at ClinicalTrials.gov before database lock, and no post-hoc modifications to the primary analysis are allowed.

**Primary model specification:** The primary model for H1 and H2 is a pre-specified multivariable logistic regression with binary treatment success (success/failure) as the outcome, incorporating five predictors: skeletal muscle density (continuous standardized z-score), VAT area (continuous standardized z-score), age (continuous), sex (binary), and baseline MWD (continuous). SMI and SAT are excluded from the primary model; they are included only as covariates in the pre-specified secondary expanded model. This model specification is fixed and will not be altered once data collection begins.

The two co-primary hypotheses (H1, H2) are tested at a Bonferroni-corrected significance threshold of p < 0.025 per hypothesis (family-wise α ═ 0.05). Hypothesis 3 (H3), pertaining to sarcopenic obesity, is classified as a secondary/descriptive hypothesis and is not subject to family-wise correction. Secondary Spearman correlation analyses between continuous body composition parameters and secondary functional outcomes will be reported with Benjamini-Hochberg false discovery rate correction (q < 0.05).

**Continuous predictor modeling**: In the primary model, skeletal muscle density and VAT area are utilized as continuous standardized z-scores. The nonlinearity of each continuous predictor is assessed using restricted cubic splines, with three to five knots selected based on the Akaike Information Criterion. Exploratory cutpoint derivation employing the Youden index (implemented via cutpointr) is conducted as a separate secondary exploratory analysis and is clearly labeled as hypothesis-generating; derived thresholds are not employed in the primary predictive model.

Internal validation is performed using bootstrap resampling (1000 samples), yielding optimism-corrected area under the curve (AUC), calibration slope, and calibration-in-the-large. The bootstrap resampling procedure encompasses the entire analytical pipeline, including nonlinearity assessment (spline knot selection); the same bootstrap samples are utilized for optimism correction across the full pipeline. For any exploratory cutpoint derivation, the derivation step is nested within a distinct independent bootstrap loop to prevent double-counting of optimism. A four-predictor model excluding baseline MWD (skeletal muscle density, VAT area, age, sex; EPV approximately 10) is included as a pre-specified robustness check (Table 3, sensitivity analysis #7a). A three-predictor model excluding sex (EPV approximately 13–14) is also part of the pre-specified robustness checks. For H3, the four-group sarcopenic obesity classification is incorporated as a categorical predictor in a similar model; H3 analyses are reported descriptively and interpreted as exploratory.

**Pre-specified secondary expanded model:** A secondary expanded confounding model is pre-specified, adjusting for the five primary predictors along with additional variables: ABI (continuous), smoking status (categorical), diabetes mellitus (binary), Clinical Frailty Scale score (ordinal), TASC II classification (categorical), SMI (continuous standardized z-score), and SAT area (continuous standardized z-score). This expanded model comprises approximately 12 predictors and, with 40 expected events, yields an EPV of approximately 3–4; this EPV limitation designates the expanded model as a robustness sensitivity analysis rather than a primary inferential tool (Table 3, sensitivity analysis #14). Results will be reported with 95% confidence intervals and interpreted cautiously due to limited EPV.

**Multiple imputation specification**: Missing data are addressed through multiple imputation by chained equations (MICE). All analysis variables—including the primary composite outcome, all five primary predictors, all covariates in the expanded model, and secondary outcomes—are incorporated into the MICE model. Twenty imputation datasets are generated (50 iterations per dataset), and estimates are pooled according to Rubin’s rules. Imputation diagnostics include convergence trace plots and comparisons of observed versus imputed variable distributions. A complete-case sensitivity analysis is pre-specified (Table 3, sensitivity analysis #15). For follow-up data potentially deviating from the missing-at-random assumption, pattern-mixture models and tipping-point analyses are pre-specified.

Cumulative incidence functions are reported alongside cause-specific hazard analyses. Pre-specified sensitivity analyses are detailed in Table 3. Component-separate analyses of the functional improvement component (excluding the revascularization criterion) and the revascularization-free survival component are designated as mandatory pre-specified secondary analyses (Table 3), not optional sensitivity analyses; these analyses isolate each failure mode and will be reported in the primary publication regardless of the primary composite outcome results.

Classification thresholds for the primary predictors (skeletal muscle density for H1; VAT area for H2) will be derived prospectively within this cohort and treated as pre-specified study outcomes rather than assumptions. For the binary primary composite outcome, optimal cut-points will be identified by maximizing the Youden index (J = sensitivity + specificity -- 1) using the cutpointr R package; 95% confidence intervals will be obtained via 1000-sample bootstrap resampling. Separate derivations will be conducted for skeletal muscle density (single threshold) and VAT area (sex-stratified: men and women analyzed independently to account for dimorphic adipose tissue distribution). For the secondary time-to-revascularization outcome, optimal cut-points will also be identified by maximizing the log-rank statistic via survminer::surv_cutpoint(), providing a time-to-event-appropriate complement to the Youden-index approach. Each threshold derivation will report the optimal cut-point value, Youden index, sensitivity, specificity, and 95% bootstrap CI. As thresholds will be derived from the same cohort used for evaluation, internal optimism is anticipated and explicitly acknowledged. Thus, all threshold-based classification results will be interpreted within the derivation-cohort framework, with external validation in an independent PAD multicenter cohort as the pre-specified mandatory next step prior to clinical translation. Literature-derived thresholds from non-PAD populations will be retained as pre-specified sensitivity analyses (Table 3) to facilitate transparent cross-referencing with existing oncological and metabolic literature.

### Additional measurements

The Clinical Frailty Scale (CFS) and Short Physical Performance Battery (SPPB) will be administered at T0 and T1 as auxiliary variables for multiple imputation and as potential confounders in robustness analyses; they are not considered primary predictors. A fasting metabolic panel (including fasting glucose, glycated hemoglobin [HbA1c], lipid profile, and high-sensitivity C-reactive protein [hsCRP]) will be obtained at T0 and T1. Serum samples for exploratory biomarker analyses (including adiponectin, leptin, and interleukin-6 [IL-6]) will be stored at --80^∘^C from all study participants at T0 and T1 for batched analysis. Assays will be performed after the first 40 participants complete follow-up (interim descriptive report) and on the full cohort at study closure. No formal stopping rule or Phase 2 decision gate applies; these analyses are exploratory and will be reported as such.

### Data management

Clinical and outcome data are captured and managed within the institutional electronic data management system, utilizing structured data entry forms, automated range validation rules, and comprehensive audit trails. Participant data are pseudonymized at the point of entry; the linkage key is maintained separately by the principal investigator and is accessible only to authorized study personnel. All data handling adheres to applicable national data protection legislation and the General Data Protection Regulation. Weekly completeness reviews are conducted by the designated data manager. Source document verification is performed on a random 10% sample of participant records, targeting an error rate of less than 2%. All outcome assessors complete structured training with a minimum passing threshold of 90% and undergo annual recertification.

## Discussion

The central methodological premise of this study posits that routine diagnostic CTA —already part of standard anatomical assessment for symptomatic PAD patients considered for revascularization—contains quantitative metabolic information that is typically disregarded in clinical practice [2, 15]. By extracting L3 body composition data from existing imaging using an automated neural network, this protocol imposes no additional burden on patients while yielding metabolic predictors with established biological relevance.

It is crucial to explicitly state a critical point regarding the scope of this study. L3 CT body composition measures systemic metabolic reserve—reflecting the whole-body distribution and quality of skeletal muscle and adipose tissue compartments—and does not directly quantify ischemia-specific myopathology in the affected lower limb musculature. The predictive hypothesis is based on two complementary mechanisms: (a) L3 skeletal muscle density reflects systemic mitochondrial function and intramuscular lipid infiltration, which constrain aerobic adaptation capacity independently of localized ischemia; and (b) VAT area quantifies a systemic pro-inflammatory endocrine compartment, whose adverse effects on exercise metabolism are well characterized across cardiovascular populations [14]. These mechanisms operate systemically rather than being limb-specific. Their relationship to PAD-specific outcomes will be delineated and bounded by the derivation cohort data; localized thigh or calf CT may provide complementary information and is pre-specified for future validation studies.

The growing body of evidence supporting L3-based opportunistic phenotyping in cardiovascular populations facilitates methodological translation to this context. Our group has demonstrated that AutoMATiCA-derived L3 parameters independently predict overall survival following TAVI [16] and has developed a user-friendly clinical implementation tool based on the same methodology [17]. The direct parallel with SET —an intervention whose outcome significantly depends on patients’ physiological reserve for aerobic adaptation—provides mechanistic justification for this approach in PAD. The broader context of opportunistic CT phenotyping programs [28, 29] underscores both the scientific precedent and the scalability of zero-burden body composition extraction from routine vascular imaging.

The composite primary endpoint is designed to capture the two clinically relevant failure modes of SET: failure to achieve meaningful functional improvement and progression to revascularization before completing the program. Functional improvement thresholds are anchored to absolute improvement benchmarks for treadmill-based SET documented in the 2019 AHA scientific statement on optimal exercise programs for PAD [19], representing the minimum criteria for a meaningful clinical response. Revascularization events are independently adjudicated and classified to facilitate sub-analyses restricted to guideline-concordant intervention decisions [4], addressing concerns that elective revascularization may be influenced by patient preference rather than objective clinical failure. The Fine–Gray competing risks model is employed for time-to-event analyses to appropriately account for the competing event of death in this elderly and comorbid population [30], thus avoiding biases that could arise from standard Cox regression in the presence of significant competing risks.

The statistical framework is calibrated to the explicit derivation role of this study. With an expected 40 treatment failures, inferential precision for model discrimination will be limited, and all conclusions will therefore be confined to the direction and magnitude of predictor associations [26, 27]. This study lacks the event volume necessary to establish validated, externally generalizable clinical decision thresholds, and any such interpretations would be premature. Bootstrap internal validation will estimate optimism-corrected model performance; however, internal validation cannot substitute for external replication in an independent cohort.

Strengths of this protocol include: a fully guideline-concordant SET program adhering to the AHA 2019 specifications [19]; a composite primary endpoint with minimal clinically important difference-informed thresholds and pre-specified sensitivity analyses; appropriate competing risks methodology; Bonferroni correction for two co-primary hypotheses (H1 and H2); bootstrap internal validation encompassing the entire analytical pipeline, including spline assessment; MICE with pattern-mixture sensitivity analyses for missing data; prospective trial registration; and adherence to Transparent Reporting of a multivariable prediction model for Individual Prognosis Or Diagnosis (TRIPOD) [31] and Strengthening the Reporting of Observational Studies in Epidemiology (STROBE) [32] reporting standards. Furthermore, an opportunistic, zero-burden imaging approach is directly scalable to clinical practice.

Limitations must be stated with equal clarity. This is a single-center derivation cohort; generalizability to other health systems, exercise program models, and patient populations cannot be assumed. The L3 vertebral level does not directly capture ischemia-specific lower limb myopathology, and the biological distance between L3 and the affected musculature is acknowledged as a limitation rather than a design strength. No PAD-specific HU threshold for myosteatosis has been prospectively validated; the cohort-derived threshold established in this study is exploratory and hypothesis-generating (see sensitivity analyses #8 and #9 in Table 3 for assessment of alternative literature-derived values of 33 HU and 41 HU, respectively). AutoMATiCA was validated in oncology and critical care cohorts [20], but not in PAD; vascular calcification—highly prevalent in PAD—represents a specific source of measurement error: calcified plaque within or adjacent to the muscle compartment may not be fully excluded by the 150 HU ceiling, potentially inflating skeletal muscle density estimates; this systematic bias is specific to PAD imaging and is acknowledged as a limitation that cannot be fully quantified without PAD-specific validation data. The performance of the segmentation algorithm in the context of PAD-specific imaging protocols has not been independently established. The three-month primary endpoint may not capture delayed functional divergence between metabolic phenotype groups. The observational design precludes causal inference. The TASC II anatomical disease severity classification is recorded as a baseline variable and is included as a covariate in the expanded confounding model; however, as a potential confounder linking disease anatomical extent to both body composition phenotype and SET response, residual confounding by vascular disease extent cannot be fully excluded in a derivation cohort of this size. Requiring an existing diagnostic CTA introduces a selection bias: patients without prior CTA workup are excluded, meaning the cohort reflects those who have undergone imaging-based revascularization evaluation. Transportability to settings without routine CTA-based PAD workup or to patients managed without imaging evaluation cannot be assumed and necessitates specific consideration in external validation studies.

Future research priorities are as follows. First, multicenter external validation of the predictor model derived here is the immediate next step. Second, a mechanistic comparison of L3 versus thigh and calf CT within the same PAD cohort would clarify the respective contributions of systemic and local body composition phenotyping to outcome prediction. Third, if predictor associations are confirmed through external validation, a randomized controlled trial of metabolic phenotype-guided versus anatomy-guided treatment selection is warranted to determine whether body composition-stratified management improves clinically meaningful outcomes.

## Conclusion

Outcome heterogeneity in SET for intermittent claudication remains a clinically unresolved challenge: a substantial proportion of patients either do not complete the SET program or fail to achieve meaningful functional benefit or require revascularization within three months, yet no validated pre-therapy metabolic predictor exists to identify these individuals. This prospective observational study aims to test the hypothesis that L3 CT-derived skeletal muscle density and VAT area—extracted opportunistically from routine diagnostic CTA at zero additional patient burden at baseline—will independently predict three-month SET outcomes in Fontaine IIa–IIb claudicants. If the findings from the derivation cohort support the primary hypotheses, and if those findings are subsequently confirmed in external multicenter validation, metabolic phenotype-guided patient selection could enhance treatment efficiency, reduce avoidable rehabilitation failure, and facilitate more rational integration of SET and revascularization pathways. This protocol constitutes the derivation phase; external validation is the essential next step before clinical translation. All thresholds, phenotype categories, and discrimination estimates reported from this study are derived from the cohort findings. Clinical translation requires prospective testing in an independent external multicenter cohort before any application to patient selection or treatment stratification. The findings of this study should not be generalized to clinical practice or patient populations that differ substantially from this single-center Czech cohort without formal external validation.

**Dissemination:** The results of this study will be submitted for publication in a peer-reviewed journal and presented at relevant national and international scientific conferences; no publication restrictions have been imposed by the funders.

## Supplemental data

Supplemental data are available at the following link: https://www.bjbms.org/ojs/index.php/bjbms/article/view/14062/4176.

## Data Availability

Anonymised data available upon reasonable request to the corresponding author following primary outcome publication.
